# National Association for Children of Alcoholics

**Published:** 1997

**Authors:** Sis Wenger

**Affiliations:** Sis Wenger is the executive director of the National Association for Children of Alcoholics in Rockville, Maryland. She also is an adjunct associate professor in the addictions studies department, University of Detroit Mercy, Detroit, Michigan, and was founder and, for 18 years, manager of Henry Ford Health System’s Maplegrove Community Education and Children’s Programs serving metropolitan Detroit

**Keywords:** children of alcoholics, organization, familial alcoholism, prevention through information dissemination, prevention through education, education and training, curriculum, school based prevention, prevention program, health care worker, workplace based prevention, outreach

No child of an alcoholic (COA) should grow up in isolation and without support. Following this dictum, the National Association for Children of Alcoholics (NACoA) — assisted by a consortium of private foundations, donors, and Federal programs and in collaboration with other organizations — advocates for “children and families affected by alcoholism and other drug dependencies.”

NACoA evolved after two groups of professionals from across the country met twice during 1982 to share their concerns, knowledge, and experiences regarding COA’s. One group included clinicians concerned with the needs of adults whose mental health problems stemmed from a childhood in an alcoholic family. The other group included counselors and social workers who primarily worked with young children experiencing a broad range of problems in families with parental alcoholism. The 22 physicians, psychologists, social workers, and educators who attended these meetings concluded that a national membership organization was needed to identify and address the unique problems of COA’s and to provide them with a means through which to voice their concerns. Consequently, NACoA was established. Now, more than 15 years later, the organization continues to focus on COA research, service provision, advocacy, and training issues as well as to address the developmental problems that COA’s may experience.

## Serving as COA Advocate

To meet the unique needs of COA’s, NACoA emphasizes the importance of providing services to them early in their lives by using the most appropriate professionals and systems. In addition, the organization advocates research on the most effective interventions. Both components are crucial to breaking the family transmission of alcoholism and preventing related behavioral health problems.

## Fulfilling the Organization’s Goals

To fulfill its mission as a COA advocacy organization, NACoA and its members are dedicated to accomplishing the following goals: raise public awareness; advocate for appropriate biomedical and psychological research as well as for appropriate education and prevention services; provide education and training to clinicians, educators, health professionals, clergy, and other people who can positively affect the lives of young, high-risk COA’s and adult COA’s in need; and develop — as well as assist other groups and individuals in developing — appropriate materials to support education, training, and effective services for COA’s.

### Educating and Training Professionals

In 1984 NACoA began hosting annual national and regional training conferences featuring researchers, clinicians, and prevention professionals experienced in COA issues. In the years that followed, several thousands of teachers, social workers, psychologists, and other professionals attended. With the assistance of other organizations (i.e., the National Institute on Drug Abuse, the National Institute on Alcohol Abuse and Alcoholism, the National Institute on Mental Health, and the Center for Substance Abuse Prevention), NACoA also initiated and cosponsored the conference “The Role of Resilience in Drug Abuse, Alcohol Abuse, and Mental Illness” in December 1994.

The results of these conferences have been both educational and therapeutic: During the mid-to-late 1980’s, for example, a wave of awareness and recovery for adult COA’s swept the United States, spawning both a wide range of mental health services and the nationwide development of Adult Children of Alcoholics self-help meetings, most notably Al-Anon. In addition, representatives of Native American communities throughout the United States who attended several NACoA national conferences were inspired to create their own organization — the National Association of Native American Children of Alcoholics (NANACoA) — to respond to the special needs of Native Americans while acknowledging the strengths of their culture and family structure. Headquartered in Seattle, NANACoA serves native communities in both the United States and Canada.

### Providing Education, Early Intervention, and Prevention Services and Raising Public Awareness

For young COA’s, early intervention and prevention services through school-based, student assistance programs have been the primary vehicle for education and support. In the late 1980’s, the U.S. Department of Education distributed NACoA’s “It’s Elementary” program materials for administrators, teachers, counselors, and students to 50,000 elementary schools. This project was the beginning of classroom education and other school-based training and support for COA’s.

In addition, NACoA has developed various educational publications and materials targeting both young and adult COA’s, parents, therapists, and educators, including an association newsletter; a COA factsheet; information kits; as well as the book *Children of Alcoholics: Selected Readings*, the first volume in a projected series. NACoA also has assisted the National Association of Broadcasters in developing an informational booklet on COA’s for its 10,000 radio and television stations and worked with the Community Anti-Drug Coalitions of America to develop its November 1997 technical assistance manual to help local coalitions address the needs of COA’s in their communities.

To further promote its cause and fulfill its mission, NACoA established the annual Margaret Cork Award in 1985 to honor pioneers working in biomedical or psychosocial research, programmatic evaluation, innovative programs for hard-to-reach COA populations or minority COA’s, or for persons developing and implementing effective early prevention programs. (Margaret Cork, a researcher with the Addiction Research Foundation in Toronto in the 1960’s and 1970’s reported her findings on COA’s in *The Forgotten Children*, the first book about COA’s to be published in North America.) Recent award recipients include Marc A. Schuckit, M.D. (1996), frequent contributor to *Alcohol Health & Research World*, for his 25 years of alcohol research, which has contributed to a clearer understanding of the family transmission of alcoholism; Alcoholics Anonymous (1995), for its 60 years of helping recovering alcoholics, many of whom are parents, return to their families and children; and Jeannette Johnson, Ph.D. (1994), for her dedication to developing an understanding of resilience in the lives of COA’s.

In turn, NACoA has received several awards for its commitment in meeting the needs of COA’s, including the National Council on Alcoholism and Other Drug Dependencies (NCADD) Humanitarian Award and a Special Commendation from Louis Sullivan, former Secretary of the U.S. Department of Health and Human Services.

As COA advocate, NACoA works with various associations and has created an affiliate system to assist organizations that focus on either providing services to children and families affected by alcoholism or expanding educational and advocacy efforts for protecting at-risk children. For example, during 1989–90, NACoA coordinated the COA Education Coalition and in 1994 was a founding member of the National Drug Prevention League. NACoA is represented on the Steering Committee of the National Leadership Forum (a group of concerned organizations, researchers, and advocates supporting research and treatment for alcoholism and other drug dependence) and is a national partner of Communities Against Drug Abuse Coalitions of America. Other NACoA associates include local affiliates of NCADD, a national family violence organization, a leading national parent educational organization, and State and local COA-focused groups.

To establish closer links with other private sector and Federal organizations interested in research and prevention services, NACoA moved its national headquarters to Washington, D.C., in 1992. At that time, the organization also narrowed its focus more specifically to children and established an active Board of Scientific Advisors, who develop and keep current the NACoA factsheet, review materials on request, write support papers, and provide consultation for NACoA initiatives, as needed.

Since its reorganization, NACoA has launched a multi-faceted Pediatric/Adolescent Medicine Initiative, which, to date, has developed Core Competencies for Health Care Providers in the Protection of Children of Substance Abusing Parents. The Initiative Panel, which includes leaders from major pediatric, adolescent medicine, and family practice organizations, has submitted the Core Competencies to multiple medical and other health care professional groups for study and adoption.

NACoA has also recently implemented both a Faith Community Initiative and an Educational Initiative. These long-term projects address multilevel issues and strategies, develop research-based programs and materials, and involve experts in each project phase.

### Promoting Research

As well as emphasizing service provision, NACoA promotes research on the most effective interventions and is investigating the correlation between alcohol use and violence and the developmental impact on children who are abused, who witness family violence, or who live in families affected by both alcoholism and family violence.

Current research indicates a possible correlation between prenatal alcohol and other drug exposure and various behaviors that may impede young children from performing successfully in school. This finding has encouraged NACoA to address questions that may lead to earlier identification of prenatally exposed children and to the development of intervention strategies to help prevent or diminish developmental, learning, and behavioral problems that may have resulted from that exposure. An interdisciplinary panel of experts from the medical field, research, special education, and the courts met in December 1996 with support from the Center for Substance Abuse Prevention to discuss the topic and to determine whether childhood problems in school resulted from prenatal alcohol exposure, a developmentally damaging environment, or a traditional learning disability. The panel recommended that studies be conducted to determine the relative effects of rearing environment on cognitive and physical defects; to relate specific areas of brain dysfunction to specific behavioral and learning deficits; and to survey, review, and evaluate the effects of prevention and intervention methods. The panel also emphasized the need to support multi-disciplinary centers that are evaluated in a multisite study of the effects of different family, social, and economic situations on a developmental level.

Because COA’s will need a voice for their concerns until alcoholism is conquered, NACoA confirms its commitment, as a national membership organization, to the role of advocate for “all children and families affected by alcoholism and other drug dependencies.”

## Available Resources

Copies of NACoA’s publications, factsheet, position statements, and educational training videos and slides are available either free of charge or for a nominal fee. COA’s, adults, students, researchers, parents, or clinicians seeking information or assistance regarding COA’s can call NACoA’s toll-free number (1–888–554–COAS) or visit the NACoA World Wide Web site (www.health.org/nacoa/).

